# Interprofessional Collaboration in Complex Patient Care Transition: A Qualitative Multi-Perspective Analysis

**DOI:** 10.3390/healthcare11030359

**Published:** 2023-01-27

**Authors:** Franziska Geese, Kai-Uwe Schmitt

**Affiliations:** Academic-Practice-Partnership, School of Health Professions, Bern University of Applied Sciences, 3008 Bern, Switzerland

**Keywords:** complex patient, care transition, interprofessional collaboration, integrated care, qualitative research

## Abstract

Healthcare professionals often feel challenged by complex patients and the associated care needs during care transition. Interprofessional collaboration (IPC) is considered an effective approach in such situations. However, a fragmented healthcare system can limit IPC. This study explored experiences of Swiss healthcare professionals regarding complex patient care transition and the potential of IPC. Professionals from nursing, medicine, psychology, physiotherapy, dietetics and nutrition, social service, occupational therapy, and speech therapy were included. A qualitative between-method triangulation design was applied, with two focus group discussions and ten individual interviews. The combination of different data-collection methods allowed us to explore complex patient care transition and to systematically add perspectives of healthcare professionals from different care settings. Three main themes were identified: (1) Participants described their vision of an ideal complex patient care transition, i.e., the status they would like to see implemented; (2) participants reported challenges in complex patient care transition as experienced today; and (3) participants suggested ways to improve complex patient care transition by IPC. This study highlighted that healthcare professionals regarded IPC as an effective intervention to improve complex patient care transition. It emerged that sustainable implementation of IPC across care organizations is currently limited in Switzerland. In the absence of strong and direct promotion of IPC by the healthcare system, professionals in clinical practice can further promote IPC by finding hands-on solutions to overcome organizational boundaries.

## 1. Introduction

In high-income countries, patient complexity is associated with chronic disease and multi-morbidity in an aging society [1,2]. Complex patients are characterized by unstable trajectories of their chronic and multiple illnesses and the need for timely and effective care coordination between healthcare professionals and care settings [3]. To provide good quality care in the context of complex patients, ‘complexity’ must be understood as a comprehensive concept with wide-ranging, interacting dimensions and varying interpretations by healthcare professionals [4]. Different complexity models have been developed, such as the ‘Theoretical Vector model’ [5] and the ‘Complexity Framework’ [1], to try to include patient-centeredness by considering the dynamics of complexity and the interaction between medical and non-medical issues with the aim of increasing health care professionals’ understanding of the service delivery necessary for complex patients [6,7,8]. Nevertheless, a common understanding of ‘complexity’ is still lacking in healthcare due to the fluctuating and dynamic state of and the changes in the illness trajectory [9]. This limited understanding is also reflected in clinical practice when healthcare professionals focus solely on the illness, the medical therapy, or the family’s socio-economic status, without considering the interaction of such health determinants, and therefore do not work collaboratively [10]. A definition for complexity in the context of the dynamically evolving patient’s needs and demands is required to allow healthcare professionals to understand this complex environment.

Different approaches were reported as effective solutions in the context of care transition for complex patients between primary care (care provided by a generalist, e.g., a General Practitioner), secondary care (care provided by a specialist such as a cardiologist or an oncologist in a general hospital), and tertiary care settings (care provided by a higher level of specialization, e.g., in a university hospital) [11,12]. Integrated care models seem promising with regard to improving patient experiences and the collaboration between healthcare professionals in care organizations, and preventing fragmented care by better addressing the care demands that arise when transitioning between settings [13,14]. Providing care continuity and a constant point of contact as part of an integrated approach seem beneficial when the state of illness fluctuates and necessitates timely care coordination [15,16,17]. However, there is evidence that, in the main, integrated care programs are implemented by region and not, e.g., as part of a healthcare system strategy [18]. The project cHaNgE (Clinical practice-oriented cHange solutions towards Active aNd healthy aGEing), for example, focused on healthy and active aging of persons with a non-communicable disease and aimed at identifying barriers inhibiting integrated care provision. Common barriers were found to be the gap of case managers and care coordinators, the lack of evidence-based guidelines for patients with multi-morbidity, inadequate training of health professionals with respect to complex patient care, and challenges in applying universal integrated healthcare solutions across Europe [13]. Language barriers were also shown to have negative impact; a nationwide cross-sectional study in Switzerland, a multilingual country, highlighted that sharing information between healthcare professionals can be inhibited due to a lack of language skills [19]. Mabire et al., in addition, explored the benefits of integrated nursing discharge planning in older medical in-patients in Switzerland and found that not all patients benefit from the integrated initiative. Here, a risk-stratified approach of the patient population was suggested to identify individuals who need an integrated nursing intervention [20].

Effective interprofessional collaboration (IPC) is understood as another complementary intervention to increase team dynamics and improve the patient’s experience of care [21]. IPC occurs when multiple healthcare professionals from different professional backgrounds provide services by working with patients, their families, carers, and communities to deliver the highest quality of care across settings [22]. Several reviews summarizing the latest evidence about the effectiveness of IPC reported favorable outcomes, such as increased quality of care, better care continuity, improved patient satisfaction, team functioning, and job satisfaction among healthcare professionals [21,23,24]. However, variability was identified between the interprofessional interventions and their outcomes, which leads to the assumption that interprofessional interventions were not well conceptualized [21]. Therefore, in order to refine the conceptual framework of IPC, a description of relevant activities was added concerning the fluctuating and dynamic state of complex patients’ needs and demands. Activities were defined as: ‘collaboration’, divided into consultative collaboration and collaborative partnership; ‘coordination’, performed as coordinated collaboration, delegative coordination, and/or consultative coordination; and ‘networking’ [25]. Schmitz et al. who qualitatively explored the perspective of Swiss healthcare professional on IPC described similar results. The authors differentiated three forms of collaboration (1) coordinative (refers to the interweaving of clearly defined, institutionalized patterns of action and learned skills of professions), (2) co-creative (collaboration of different professional and individual skills over relatively long periods of time), and (3) project-like collaboration which can be placed in the middle of a continuum ranging from coordinative to co-creative collaboration as a form of ad hoc collaboration [26]. To implement IPC sustainably in a healthcare system inhibiting factors have to be understood. A systematic meta-review by Wei et al. [27] summarized influencing factors on organizational, team, and individual levels based on studies performed in 14 countries, excluding Switzerland. The authors reported that outcomes for patients, healthcare professionals, and the organization can be negatively impacted when those influencing factors are not addressed. For example, a common definition of organizational values and mission, allocated power and supportive structures can improve IPC, whereas the lack of these factors can decrease IPC. The same applies to factors such as role clarity, conflict management, and leadership at the team level, as well as communication, trust, and respect at the individual level [27].

In summary, the evidence with respect to factors that are influencing IPC is largely based on different quantitative international studies whereas qualitative studies addressing the perspective of health professionals working in the care of complex patients is missing. Specifically, for Switzerland, corresponding analyses are missing. Just one qualitative study was identified that examined the views of health professionals of IPC in Switzerland [26]. However, this study refers to the general nature and challenges of IPC but is too nonspecific regarding complex patient care. Accordingly, this study aimed to explore complex patients’ care transition and the potential of IPC to improve such transition from the perspectives of different healthcare professionals.

## 2. Materials and Methods

### 2.1. Design

A qualitative between-method triangulation design was applied to explore the provision of complex patient care transition from the different perspectives of different health professions [28]. The COREQ checklist (Consolidated criteria for Reporting Qualitative research) [29] served as the basis for describing the study. The research team consisted of two researchers (one PhD in biomedical sciences, one MSc in nursing) and two research assistants (BSc Nursing, BSc Nutrition). The triangulation of qualitative methods was chosen to systematically add perspectives and thus to gain knowledge about the transition of complex patient care and the relevance of IPC [28,30]. 

### 2.2. Sample and Setting

Healthcare professionals working in primary, secondary, and tertiary care settings in Switzerland were the targeted sample of this study. Healthcare professionals were included when they had graduated in a health profession such as nursing, medicine, psychology, physiotherapy, dietetics and nutrition, social service, occupational therapy, or speech therapy and when they were still actively providing care services. Healthcare professionals were excluded when they were unable to speak and understand German, had less than two years’ experience working in healthcare, and had worked for less than 6 months at the same institution. 

The recruitment of study participants followed a multi-sampling strategy and a stepwise approach based on the data-collection process. First, the authors used a pragmatic approach by contacting medical and nursing leaders of their network asking to suggest or provide, respectively, participants for the interviews who match the inclusion and exclusion criteria. These leaders were responsible for the medical disciplines of psychiatry, palliative care, and orthopedics of an affiliated university hospital in Switzerland that partners with the institutionalized Academic-Practice-Partnership of a University of Applied Sciences in a German-speaking canton of Switzerland, where the authors are employed. This Academic-Practice-Partnership aims to support clinical partners to identify care issues and to facilitate research to provide clinical recommendations for good quality care. These disciplines were chosen based on their public health relevance, as identified by the Swiss Federal Office of Public Health [31], and on the clinical experience of the authors’ research group that complex patients often have non-communicable or psychiatric illnesses that are treated in these disciplines. Within these disciplines, the healthcare professionals forming our convenience sample were invited to participate in the study to represent views from a tertiary care setting. In a next step, these participants were asked to recommend further participants from primary and secondary care settings. In a third step, national associations representing health professions were invited to identify a delegate to participate in this study and thus to contribute the views of the association in the context of complex patient care transition.

Additionally, three patients and caregivers diagnosed with chronic and/or multiple illnesses or caring for such a person were involved in this study to contribute their individual experiences. The patients and caregivers were recruited through an established so-called ‘user group’ of a university hospital; they are trained to be involved in and support research projects. This participatory approach is recommended by the World Health Organization [32] to better include patients’ and caregivers’ views. 

### 2.3. Data Collection

#### 2.3.1. Development of an Interview Guide

A semi-structured interview guide was developed, following the guide produced by Kallio et al. [33], with open-ended questions encouraging participants to share their experiences and perceptions. An initial literature search regarding complex patient care transitions identified topics that were included in the interview guide. These topics encompass factors influencing complex patient care transitions [34], personal experience regarding such care transitions [35,36], and the potential of IPC in this context [21,37]. The interview guide was validated by a research team member (K.-U.S.) before its first use. Due to the different perspectives explored here, relevant new topics were continuously added to the interview guide to further deepen the knowledge gained. The main interview questions were: (1) Where do you see a need for optimization in the care transition of complex patients with regard to continuity of care and what is the reason for this? (2) How could the care transition of complex patients be improved with regard to continuity of care? (3) What would you recommend to improve the care transition of complex patients? (4) To what extent could IPC contribute to the optimization of the care transition for complex patients?

#### 2.3.2. Focus Group Discussions

Two focus group discussion sessions were conducted by three members of the research team (F.G., K.-U.S., S.S.) to explore the experiences of healthcare professionals regarding complex patient care transition from primary, secondary, and tertiary care settings. It was aimed to include 4 to 8 interviewees in each discussion round representing different healthcare professions, and in the second discussion round different care settings should be represented. The first session included two users and six healthcare professionals working in a university hospital, i.e., a tertiary care setting. A second focus group discussion session was then held with 1 user and 12 healthcare professionals from primary and secondary care settings; 4 of those participants were also working in a hospital. The aim was that the exchange of experiences would generate ideas on how to address relevant issues in care transition and would also increase the awareness of professionals working in a different care setting. The focus group discussions took 120 min each and were audio-recorded and thematically summarized by research team members (K.-U.S., S.S., R.S.), who also made notes during these sessions. At the end of each session, all themes were summarized using the mind-mapping technique and the contents were summarized, checked and validated by the interview participants [35]. A written summary of each session was prepared (K.-U.S., R.S.) and used for data analysis.

#### 2.3.3. Individual Interviews

The individual interviews took place after the focus group discussions. The aim was to conduct 8 to 10 additional interviews. The healthcare professionals were interviewed by telephone to gain further and more personal insights that might not always be given in focus group discussions. As a preparatory step, the results of the two focus group discussions were summarized by the research team (F.G., K.-U.S.) and sent to the individual interview participants. Participants then discussed the results in a semi-structured way together with the interviewer (F.G.). The interviewees were asked if they agreed with the results and/or had additional remarks. All interviews were audio-recorded and summarized by the interviewer (F.G.). 

Between December 2019 and April 2020, a total of 4 users and 34 healthcare professionals were invited to take part in this study to share their experiences. Of these, 3 users and 25 healthcare professionals eventually participated in one of the focus group discussions or individual interviews. The remaining invitees did not respond at all or did not find time to participate due to the COVID-19 pandemic.

### 2.4. Data Analysis

Qualitative data were thematically analyzed using the methodology devised by Braun and Clarke (2006) [38]. This method followed an inductive analysis process based on a constructivist understanding to recognize various perceptions of healthcare professionals and to better construct individual realities. A constructivist understanding considers the sociocultural background of healthcare professionals and structural conditions regarding the care transition process [38,39]. The thematic analysis process is comprised of an initial examination of the data material, data-guided coding, a search for themes based on codes, the critical review of the themes generated, and the final definition of the themes. 

The analysis of data was initially undertaken by one researcher (F.G.), a research associate who is an experienced nurse and care coordinator and who has experience with this research method. The software MAXQDA^®^ (versions 2020.3/2020.4, VERBI Software GmbH, Berlin, Germany) supported the organization and analysis of qualitative data. Codes were derived from the written summaries and then built into themes. Generated themes were complemented by relevant content derived from the focus group discussions and the individual interviews. The consolidation of all data material was supported by mind maps that were created after each focus group discussion/interview [38,40]. The data and emerging themes were also peer debriefed with the second author (K.-U.S.), who took notes during the focus group discussions and is familiar with the qualitative research method. Both researchers discussed the possible interpretations and reached a consensus on the final themes.

## 3. Results

**Figure 1:**
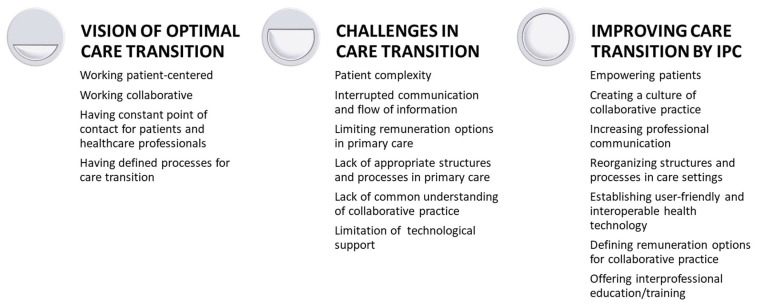
Summary of the main and sub-theme system.

Table 1 provides a description of the study participants. A total of 4 users and 25 healthcare professionals participated in this study, all were German speaking. The number of interviewees in the second focus group discussion was higher than envisaged due to a strong interest of healthcare professionals working in primary care. The analysis of qualitative data synthesized into three overarching themes: (1) Participants described their vision of an ideal care transition, i.e., the status they would like to see implemented; (2) participants reported challenges in complex patient care transitions as experienced today; and (3) suggestions on how to improve IPC were identified. Figure 1 summarizes the main themes and sub-themes.

### 3.1. Vision of Optimal Care Transition

Overall, participants shared the vision that the optimal complex patient care transition must be established based on a common understanding of the care and a corresponding care approach. Participants from nursing and medicine pointed out that a patient-centered approach is beneficial to address complex patient needs. Two participants with a nursing background added that the provision of patient-centered care requires all providers to place complex patient needs at the center of treatment and care planning. Furthermore, a remuneration system that supports a collaborative practice of healthcare providers within one institution as well as between different care organizations was recognized as an incentive for a transition process tailored to patient needs. Furthermore, participants explained that a constant point of contact is beneficial for complex patients and healthcare professionals alike to provide a timely and continuous flow of information to coordinate care and treatment. One participant working as a psychiatrist in primary care explained in this context:

*‘The most important thing is that you can exchange information in person. That is the most effective and fastest form of communication and the best guarantee that information flows. And that is of course increasingly difficult in more operationalized systems today. I experience this, for example, with referrals to inpatient psychiatry hospitals. When I fill out a referral form, I can do it very meticulously, but it also takes me 1 to 2 h. Or I know whom I can call and that is much easier for someone who is locally well-connected. I know where and whom to call and it’s done in five minutes*.’ (01_focus group discussion)

The identification and definition of the healthcare provider’s responsibilities and tasks, e.g., nutrition and medicine, were deemed particularly important in complex patient care, where patients experience various care needs. Reflecting on the responsibilities and specific tasks during the care transition process, participants stated that the traditional medical lead does not fit with the expectations in this context. One General Practitioner (GP) and a community nurse working in primary care illustrated how the traditional medical lead can be divided between two professions to better address patients and healthcare system expectations. Splitting the lead of a complex patient case into a medical responsibility, which goes with the GP, and assigning the overall responsibility for the case and care management to the community nurse ensures a more transparent process of care coordination. Most participants stated that processes relevant for an optimal care transition must be defined and be transparent to all involved healthcare professionals (i.e., defining the complex patient care transition between hospital and primary and secondary care settings, respectively). 

### 3.2. Challenges in Care Transition

A challenging care transition process was experienced with increasing patient complexity. Participants summarized that a complex patient will have an increasing number of diagnoses and when illnesses become chronic, the needs of the patient and their healthcare providers arise and can come into conflict. A participant working as a medical doctor in palliative care added that healthcare professionals are theoretically aware of complex patients, but they often do not realize when a complex care situation actually occurs:

*‘The transition to a complex patient and its care provision can be very linear. (…) In palliative care, we know that when it is a palliative situation, it is usually relatively complex. However, there is an illness trajectory long before that, that shows, that it is or becomes complex, even though you don’t think about it, because you are busy with therapies all the time.*’ (I04_individual)

Furthermore, many participants stated that limited communication between healthcare professionals and a delayed flow of relevant information characterize an interrupted complex patient care transition. Participants criticized, for example, that medical reports were tailored to health insurance remuneration schemes rather than to the needs of patients or healthcare providers. Some health professions thus implemented their own mono-professional reports without considering any interprofessional aspects. The lack of a standardized and common ground for reporting was noted as a severe limitation. Information about care coordination was often missing due to its irrelevance to health system structures, such as remuneration of coordinative and therefore collaborative practice, and due to the lack of standardized processes. Moreover, the quality of reports was questioned as well. One participant with a medical background stated that the current form of a medical report does not address the information needs of healthcare professionals with respect to an optimal complex patient care transition. This is because the inclusion of care coordination in a collaborative healthcare context is missing:

*‘(…) yes, that is how medical reporting works, due to its relevance for insurance billing. This and that should be included, but if you don’t want to extend the report to 4 pages, you leave out the rest (…) which then influences the quality of the report. The quality is catastrophic, the content is zero!’* (I04_individual).

In terms of communication, participants mentioned that, particularly in primary care, opportunities for exchange are missing between healthcare providers to plan and coordinate care in a collaborative manner. One reason for this was again identified as the lack of full remuneration for coordinative activities. One participant, working as a psychiatrist in primary care, explained that the current remuneration system encourages referral of complex patients with high psycho-social needs to psychiatric daycare organizations. Furthermore, a participant working as a psychologist explained that for his/her profession the complex patient care transition is hindered by the legislative requirement:

*‘(…) We are still perceived as assistant staff (psychologists), so according to the Swiss health insurance system we cannot reimburse services on the grounds of the basic insurance, the delegates (who receive a referral by medical doctors) can, but the self-employed cannot. (…) With the GPs, we (as self-employed psychologists) have found a common ground to collaborate; with psychiatrists, in contrast, it is partly difficult, due to their problems with the next generation (i.e., low number of psychiatrists and the number is further going down) and therefore they are under pressure.’* (I03_individual).

Participants with different professional backgrounds discussed how complex patients also experience problems, such as stressful situations, that could be treated directly by a psychologist and physical problems that could be treated by physiotherapy without a prior medical consultation. However, complex patients are dependent on their GP referral to specialists if the service is refunded by their health insurance provider.

Structures and processes of a complex patient care transition between different care organizations were criticized by participants representing primary, secondary, and tertiary care settings. A care transition between these settings was described as requiring particularly high flexibility from healthcare professionals. The structural and procedural differences between the two care settings were experienced as challenging when too different. The schedule of a GP in primary care who has appointments every 30 min does not, for example, fit with a surgeon working in hospital care who is mainly in the operating theatre. Due to the institutional structures and processes the two medical doctors can hardly reach each other, which delays the information flow and makes coordination difficult. 

Furthermore, participants explained that collaborative practice is compromised by a lack of knowledge regarding the professional background of healthcare professionals involved in complex patient care. Not being aware of the capabilities and responsibilities of other healthcare professionals was identified as a major issue. Participants with a non-medical background explained that their understanding of the role of individual professions, e.g., of medical doctors, is often experienced as hierarchical and sometimes challenging. It was explained that the professional attitude can result in a misleading understanding of collaboration and might be mirrored by the fragmented care system. Regarding this fragmentation, a participant added:

*‘What I still notice professionally and institutionally is that only where someone works or what is relevant for them is just of interest. And it is just not enough patient-tailored or related to what is required (for a collaborative complex patient care transition). And I believe that this feels often like a battle (between healthcare professionals and institutions) and thus remains in these silos.’* (I06_individual)

Digital tools for documenting patient information were described by the participants as relevant aids. However, participants’ experience was that these tools vary among care organizations and are not compatible between different settings, which limits or prevents the healthcare professional from exchanging and accessing patient reports digitally. As a result, healthcare professionals explained that they must wait until they receive a report before they can introduce the next step in complex patient care provision. The participants concluded that the current digital documentation tools are not sufficiently user-centered and are limited with respect to their ability to foster a collaborative practice. 

### 3.3. Improving Care Transition by Interprofessional Collaboration

The study participants considered the promotion and implementation of IPC as beneficial for the complex patient care transition. IPC was understood by the participants as a strong and collaborative practice between different healthcare professions based on mutual respect. IPC was regarded as the main intervention in collaborative practice next to intra-professional (between professionals from one profession) and multi-disciplinary cooperation (between different medical disciplines). To create a collaborative healthcare practice between professionals, two participants mentioned that an interprofessional attitude must be established by asking:

*‘Which information does the other healthcare provider need to continue providing care?’* (01_focus group discussion).

Participants with a non-medical background added that the complex patient must be a full member of the interprofessional team; for example, he/she should attend care coordination meetings. However, to prepare complex patients to be part of the interprofessional team and participate collaboratively, participants stated that patients must be empowered first. Additionally, it was stated by a user that complex patients with, e.g., a hearing impairment, must also be considered as a part of the interprofessional team. A possibility was seen in peer support:

*‘It was about caring for an 86-year-old woman. In some instances, she did not understand everything. Then she was embarrassed to ask the doctor again, because she was worried that people would think that she was an old woman and that she was already a bit stupid. (…) I always think that help is needed, which should be installed in the hospitals and so on (…) and stand by the side of those affected, for example through volunteers or peers.’* (01_focus group discussion)

Most participants agreed that communication between healthcare professionals and between care settings must be improved. It was recommended to implement dedicated interprofessional communication platforms in which professionals from different care settings plan and coordinate the complex patient care transition together. 

Further potential for improvement was seen in defining structures and processes within and between care organizations/settings to provide an optimal care transition. It was recommended that organizational structures and processes must be reflected upon regularly and, if necessary, adjusted to support IPC. The development of interprofessional concepts and guidelines/standards to improve the transparency of relevant processes was also mentioned. Some healthcare professionals supported this idea; some participants with a medical background hesitantly expressed a concern that the development and implementation of corresponding documents often resulted in a higher administrative workload. Poor integration of these concepts/guidelines in daily practice and consequently poor compliance were quite often experienced by medical doctors. In contrast, the use of interprofessional checklists in the context of palliative care provision was mentioned as a good example of how to foster a coordinated interprofessional complex patient care transition process. 

The participants agreed on the provision of a digital documentation tool that might support the exchange of information in a collaborative manner. On the one hand, such a system was supposed to be user- and patient-friendly, respectively, as well as interoperable. On the other hand, participants regarded it as necessary that the healthcare policy must be supportive to ensure such a documentation system is accessible to all relevant healthcare professionals. 

Overall, participants saw great potential in the implementation of a remuneration option to support the collaborative practice and to serve as an incentive to enforce IPC in the context of complex patient care transition. Participants working in primary care stated that remuneration options must be created such that all health professions can charge coordinative activities. In this context, one participant made the point that new remuneration options should be continuously monitored and adapted, if needed:

‘*This question of costs comes up again and again. And with the pressure to economize… it also helps, if you look at the issue the other way round and ask yourself, what will it cost if we (healthcare professionals) don’t do it? What if we don’t do it because of the cost? The follow-up costs that arise because of problems in the (complex patient) care transition and, if therapies are not provided… that will be much more expensive on average compared to investing in coordination services at the beginning. Nonetheless, after monitoring this alternative approach it can always be decided to change back to the old modus.’* (01_focus group discussion)

Some participants also highlighted the relevance of interprofessional education and training to foster interprofessional skills. Additionally, it was commented that the level of IPC is associated with the size of each healthcare professional’s network. Knowing who should be contacted for which task/purpose was said to foster a culture of interprofessional exchange. Thus, it was concluded that role models in practice who work on an interprofessional level are needed as best-practice examples to improve IPC.

## 4. Discussion

**Figure 2:**
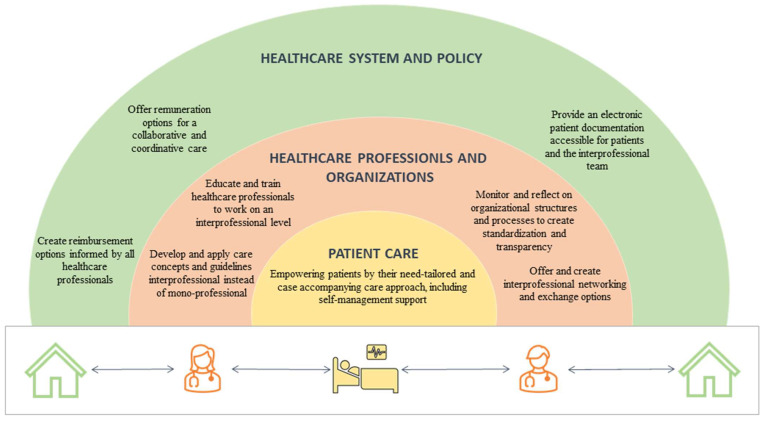
Systematic to foster IPC on patient, organizational and health system levels. Based on the needs of patients, the contributions of healthcare professionals and the requirements of the healthcare system are illustrated.

This study confirmed that care transition creates various challenges, particularly for complex patients. Overall, healthcare professionals experienced such care transitions as demanding in any care setting, be it primary, secondary, or tertiary care. Certain personal and structural factors were reported as inhibiting IPC and thus negatively influencing the care transition. Such factors were identified on the individual level of healthcare professionals, on the organizational level, and on the overarching level of the Swiss healthcare system. To optimize complex patient care transition from the perspective of users as well as healthcare professionals, the participants of this study recommended addressing these factors where IPC plays a major role. Figure 2 summarizes these factors and places them into context of how to foster IPC at each level. This study was embedded in the Swiss healthcare system, but its focus is applicable in a broader sense, placing IPC in the center of interest.

The study results highlighted that there are ambiguous processes, such as experiencing the lack of a clearly defined lead in a complex patient care transition, that occur frequently and are a major source of dysfunctional IPC. Traditionally, based on Swiss legislation, treatment and care were coordinated and organized by medical doctors. This is no longer standard today. A lack of GPs in primary care, for example, increases their burden due to higher caseloads and thus reduces their capacity to coordinate different healthcare providers [41,42]. Moreover, in secondary and tertiary care organizations’ capacities to coordinate complex patient care transitions are sparse. As a result, a dedicated case lead or a constant point of contact during the day is often missing. However, as the participants of this study stressed, both aspects are crucial for a successful care transition in complex patients. Case management or care programs provided by Nurse Practitioners in primary or hospital care aim, for example, to bridge that gap and offer cost-effective, timely care coordination and a constant point of contact [43,44,45]. However, the implementation of new, integrated care models that require an autonomous practice by advanced non-medical healthcare professionals is challenging [46,47]. Furthermore, study participants concluded that the current structure of the healthcare system is inhibiting IPC from providing good quality care in complex patient care transitions. In particular, healthcare professionals working in primary care noted that the important aspects of IPC, such as coordinative phone calls or coordination meetings, are barely remunerated by health insurance. Due to such financial limitations, healthcare professionals were reluctant to coordinate care in an interprofessional context. A legal framework that offers a remuneration system for coordinative activities during the care transition to (non-)medical healthcare professionals would contribute to a sustainable interprofessional collaborative culture [48]. Policymakers need to be informed about relevant changes in complex patient demands and requirements for healthcare professionals to offer timely and good quality care. To achieve this, the integration of a Swiss learning health system could be the first step to support the translation of evidence from clinical practice to the health policy level [49]. Furthermore, the development of a road map provided by, for instance, a professional body that describes the healthcare problem, the needs and the possible solutions could help to inform relevant decision-makers about necessary steps to discuss structural changes [50]. 

From this study, it emerged that IPC is regarded as one important intervention to improve complex patient care transition. However, promoting collaborative practice between healthcare professionals working in different care settings demands different strategies. From an educational point of view, healthcare professionals need to learn how to work on an interprofessional level. Effective approaches, such as interprofessional problem-based learning and interprofessional simulation training, could prepare new generations of healthcare professionals before transitioning to work-life [51,52]. However, continuing interprofessional education and training for all those involved is needed via different formats, such as interprofessional lectures and training at the workplace or interprofessional post-graduate programs [53]. It was shown that care organizations could promote IPC by following a top-down approach and offering interprofessional training programs, less hierarchy, and nominating role models who follow the interprofessional approach, all of which would result in improved quality of care and better staff wellbeing and job satisfaction [54,55]. IPC should therefore be understood as a multi-level intervention, with both individual and organizational components (as summarized in Figure 2). It needs to be understood that healthcare professionals, care organizations, and the structures of a health system create together an environment of collaborative practice.

### Strengths and Limitations

This study benefited from the qualitative between-method triangulation, which allowed us to add perspectives from different healthcare professionals working in a variety of care settings. The study participants offered a comprehensive insight towards understanding of complex patient care transition and the relevance of IPC in this regard. However, Braun and Clarke (2019) argue that only about 80% recurring major themes can be assumed from 8 individual interviews and 2 to 3 focus group interviews [56]. Our study with 10 individual interviews and 2 focus group discussions is in this range, i.e., full thematic saturation cannot necessarily be assumed. A large-scale qualitative study with approximately 15 interviews per healthcare profession as well as per care setting would allow for a holistic analysis exploring the potential of IPC during complex patient care transition in Switzerland. Considering participants from different parts of the country would then also be of benefit. At the time, study participants were mainly working in the German-speaking part of Switzerland, but representatives of the professional bodies were asked to discuss the study results within their teams to ensure that a national context was considered.

Additionally, the COVID-19 pandemic might have impacted the results. This study was conducted at an early local phase of the pandemic, the participants could thus hardly link the pandemic to the research topic. Hence, it is not expected that the pandemic had a relevant impact on the study results.

## 5. Conclusions

Through exploring the state of IPC in complex patient care transitions, this study highlighted that healthcare professionals regarded IPC as an effective intervention when properly implemented. It emerged that sustainable implementation of IPC across care organizations is currently limited. Successful IPC relies on individuals who act as role models and who initiate and represent a culture of IPC. Organizational leadership should establish a corresponding environment for IPC to flourish. Healthcare professionals need to acquire skills that promote collaborative practice, e.g., in the form of continuing interprofessional training. Where the healthcare system does not foster IPC, professionals in clinical practice can promote IPC by finding hands-on solutions to overcome organizational boundaries.

A healthcare system in which coordinative activities are not fully remunerated limits IPC. This can negatively impact the quality of care and might even generate additional cost. Health policy should thus ensure that structures are created to exploit the potential of IPC.

## Figures and Tables

**Table 1:** Description of study sample.

	Focus Group Discussion (1)	Focus Group Discussion (2)	Individual Interviews
Number of participants	*n* = 8	*n* = 13	*n* = 10
Professional background	- Medicine (3) - Nursing (2) - Midwifery (1) - user (2)	- medicine (3) - nursing (3) - psychology (1) - midwifery (1) - nutrition & dietician (1) - physiotherapy (1) - business administration/management (1) - user (1)	- medicine (3) - nursing (2) - psychology (1) - dietician and nutrition (1) - occupational therapy (1) - social care (1) - physiotherapy (1)
Represented care setting	- university hospital	- university hospital, - psychiatric university hospital, - community care, - General Practitioner practice, - rehabilitation clinic, - elderly and long-term care facility	
Organization/Association			- Swiss Association of Medical Doctors (FMH), - Swiss Association of Applied Psychology (SBAP), - Swiss Association of Dieticians and Nutritionists (SVDE), - Swiss Association of Occupational Therapists (EVS/ASE), - Swiss Association of Social Workers in Healthcare (SAGES), - Swiss Association of Physiotherapists (physioswiss), - Foederatio Medicorum Psychiatricorum et Psychotherapeuticorum (FMPP), - Swiss Association of Palliative Care (palliative.ch), - Swiss Association of Community Care Organization (Spitex Schweiz), - Swiss Association of Long-term Care (Langzeitpflege Schweiz)

## Data Availability

The data generated and analyzed in this study are not openly available as participants did not consent to share data with third parties, but they are available from the corresponding author on reasonable request.
